# Kidney transplantation or dialysis in older adults—an interview study on the decision-making process

**DOI:** 10.1093/ageing/afac111

**Published:** 2022-05-03

**Authors:** Tessa S Schoot, Marieke Perry, Luuk B Hilbrands, Rob J van Marum, Angèle P M Kerckhoffs

**Affiliations:** Department of Nephrology, Radboud Institute for Health Sciences, Radboud university medical center, Nijmegen, the Netherlands; Department of Nephrology, ‘s-Hertogenbosch, Jeroen Bosch Hospital, the Netherlands; Department of Geriatric Medicine, Radboud university medical center, Nijmegen, the Netherlands; Department of Nephrology, Radboud Institute for Health Sciences, Radboud university medical center, Nijmegen, the Netherlands; Department of Geriatric Medicine, ‘s-Hertogenbosch, Jeroen Bosch Hospital, the Netherlands; Department of Clinical Pharmacology, ‘s-Hertogenbosch, Jeroen Bosch Hospital, the Netherlands; Department of Elderly Care Medicine, Amsterdam University Medical Center, the Netherlands; Department of Nephrology, ‘s-Hertogenbosch, Jeroen Bosch Hospital, the Netherlands; Department of Geriatric Medicine, ‘s-Hertogenbosch, Jeroen Bosch Hospital, the Netherlands

**Keywords:** kidney transplantation, dialysis, chronic kidney disease, end-stage kidney disease, kidney replacement therapy, older people

## Abstract

**Background:**

In older patients with end-stage kidney disease (ESKD), the choice between kidney transplantation (KT) and dialysis may be more complex than in younger patients because of a higher prevalence of comorbidities and frailty. This study aims to provide greater insight into the current decision-making process by exploring the expectations, experiences and health outcome priorities of all stakeholders.

**Methods:**

We performed semi-structured interviews with patients ≥65 years with ESKD (eGFR <15 ml/min/1.73m^2^, KT recipient or treated with dialysis), patients’ relatives and healthcare professionals (nephrologists, nurses and medical social workers). Interviews were conducted until data saturation and thematically analysed.

**Results:**

We performed 36 interviews (patients *n* = 18, relatives *n* = 5, healthcare professionals *n* = 13). Thematic analysis revealed five themes. Older patients’ health outcome priorities were mostly related to quality of life (QOL). Individual older patients showed marked differences in the preferred level of engagement during the decision-making process (varying from ‘wants to be in the lead’ to ‘follows the nephrologist’) and in informational needs (varying from evidence-based to experience-based). On the contrary, healthcare professionals were quite unanimous on all aspects. They focused on determining eligibility for KT as start of the decision-making process, on clear and extensive information provision and on classical, medical outcomes.

**Conclusions:**

The decision-making process could benefit from early identification of older patients’ values, needs and health outcome priorities, in parallel with assessment of KT eligibility and before discussing the treatment options, and the explicit use of this information in further steps of the decision-making process.

## Key Points

- Many older patients with kidney failure prioritise QOL-related outcomes above outcomes like life expectancy.
- Individual older patients have different informational needs and preferred level of engagement in the decision-making process.
- Patients’ values and health outcome priorities should be explored before discussing treatment options.

## Introduction

A large proportion of the patients with end-stage kidney disease (ESKD) are older adults. In the USA, 74% of the patients treated with kidney replacement therapy (KRT) are aged 65 years or older (1). Of the two KRT modalities, kidney transplantation (KT) and dialysis, KT is generally considered superior because of a longer life expectancy and better quality of life (QOL) compared with dialysis (2–4). In 2019, 32% of the KTs in the Netherlands (±*n* = 300) were performed in patients aged 65 years or older (5).

However, KT might not be the best KRT modality for all older adults. Factors such as older age, comorbidities and frailty are associated with a higher risk of complications and mortality after KT (2, 6–9). Consequently, the choice between KT and dialysis in older adults has to be tailored to the individual situation.

Clinical guidelines regarding the choice between KT and dialysis recommend shared-decision making (SDM) (10–12), which is ‘an approach where clinicians and patients make decisions together using the best available evidence’ (13). In older adults, SDM should have a goal-oriented approach instead of a disease-oriented approach (14, 15). Thus, a key step of successful SDM in older adults is the early identification of patient values and goals of care (i.e. health outcome priorities) (14).

**Figure 1:**
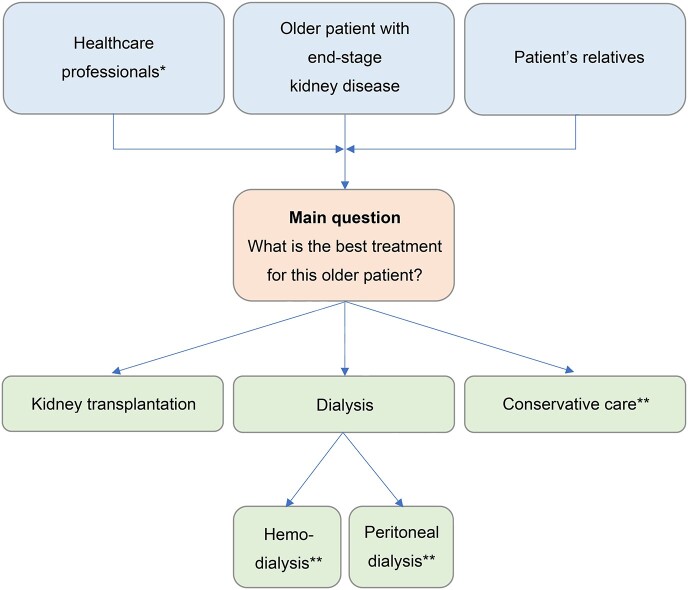
Treatment options of an older patient with ESKD, scope of this study and people that are involved in the decision-making process. This study focuses on the decision between KT and dialysis. Blue = people that are involved in the decision-making process. Red = main question of the decision making process. Green = treatment options. ^*^ Nephrologists, nurses, medical social workers ^*^^*^ Beyond the scope of this study

Figure 1 shows the treatment options of older patients with ESKD and the people who are involved in this decision-making process. Little is known about the current decision-making process regarding the choice between KT and dialysis in older adults. For example, it is unknown whether SDM occurs, or what the health outcome priorities of the older adults facing this decision are. We therefore aimed to provide greater insight into this decision-making process by exploring the expectations, experiences, values and health outcome priorities of all persons involved in this process (i.e. older adults with ESKD, their relatives and healthcare professionals, see Figure 1). The results of this study could contribute to improved (shared) decision-making regarding the choice between KT and dialysis in older adults.

## Methods

### Study design

We performed a qualitative study with semi-structured interviews. The Consolidated criteria for Reporting Qualitative Health Research (COREQ) checklist (16) was used for the reporting of this study; Appendix 1 shows the completed checklist. The institutional review boards of the Radboudumc and Jeroen Bosch Hospital approved this study. This study was conducted in adherence to the Declaration of Helsinki.

### Setting and participants

As we aimed to gain broad insight into the decision-making process, the study population consisted of all persons involved in the decision-making process in Dutch clinical practice: patients, patients’ relatives and healthcare professionals (nephrologists, nurses and medical social workers). Because we wanted to collect data about expectations (about KRT) and experiences (with the decision-making process), we included both patients who were not yet on KRT (i.e. patients currently facing the decision between KT and dialysis) and patients already treated with KRT (i.e. patients who made the decision between KT and dialysis in the past). Additional inclusion criteria for patients were ESKD (i.e. estimated glomerular filtration rate (eGFR) < 15 ml/min/1.73m^2^, dialysis treatment or KT recipient), age 65 years or older (at start of KRT) and eligibility for both KT and dialysis (at start of KRT, judged by the treating nephrologist).

Participants were recruited from two Dutch hospitals: the Radboudumc (transplantation centre with dialysis ward) and the Jeroen Bosch Hospital (hospital with dialysis ward). The standard pre-dialysis and pre-transplantation educational programmes of the participating centres are described in Appendix 2. Potential participants were approached face to face, by telephone or via email. None of the approached subjects refused or withdrew participation. To assure broad diversity in the opinions and ideas to be collected, patients and healthcare professionals were recruited using purposive sampling. We aimed for variation in patient characteristics (e.g. age, gender, current KRT modality, treatment preference) and professional characteristics (e.g. age, gender, profession, clinical experience). Relatives were recruited via participating patients. Again, we aimed for variation in personal characteristics (e.g. age, gender, relation to patient). All participants provided written informed consent.

### Data collection

The first version of the interview guide was developed by the research team (TS, AK, MP) based on an extensive literature search and clinical experience. During data collection and analysis, the interview guide was constantly adjusted based on new insights. Appendix 3 contains the final version of the interview guide.

Interviews were performed from July to December 2020 by two interviewers. Appendix 1 (domain 1) shows the personal characteristics of the interviewers. During the interviews, only the participant and interviewers were present. Interviews were performed face to face (in the hospital or at the participants’ home), or, due to the coronavirus pandemic, via video call or telephone. Interviews were audio recorded and transcribed verbatim afterwards. Interviews were conducted until interviews did not reveal new concepts and themes (i.e. data saturation). The level of data saturation was assessed during frequent research meetings (TS, LHu, MP, AK) using the ‘information power’ model. This model enables systematic reflection on five items with an impact on the information power (i.e. study aim, sample specificity, use of established theory, quality of dialogue and analysis strategy) (i.e. the more information the sample holds, the lower amount of participants is needed) (17).

### Data analysis

Interviews were analysed using thematic analysis (18), supported by ATLAS.ti (version 8.4.20). In the first phase (open coding), all phenomena in the text were labelled by two researchers (TS, LHu) separately. Next, codes were compared, and similar codes were grouped together into axial codes (TS, LHu). New transcripts were analysed using a codebook with these axial codes by two researchers (TS, LHu) separately. During this phase of axial coding, existing axial codes were rephrased and new axial codes were added if necessary. The results were discussed in frequent research meetings (TS, LHu, MP, AK). Finally, all axial codes were categorised and themes were defined.

## Results

We performed 36 interviews: 18 with patients, five with patients’ relatives and 13 with healthcare professionals. Participants were recruited from the Radboudumc (*n* = 26) and the Jeroen Bosch Hospital (*n* = 10). Interviews were performed face to face (*n* = 25), via video call (patients *n* = 2, relatives *n* = 2, healthcare professionals *n* = 2), or by telephone (patients *n* = 2, healthcare professionals *n* = 3). Mean interview duration was 42 minutes (standard deviation 11 minutes). Table 1 summarises the population characteristics, and shows that purposive sampling successfully resulted in variation in most predefined characteristics such as age and KRT modality. However, the number of participants with a high educational level among patients and relatives was limited. All participating patients were retired.

**Table 1:** Population characteristics

		Patients	Patients’ relatives	Healthcare professionals
Number of participants		18	5	13
Age (years)		71 (6)	69 (25)	46 (16)
Gender (male)		12 (67%)	1 (20%)	5 (39%)
Living situation (alone)		6 (33%)	-	-
Educational level^*^	Low	13 (72%)	4 (80%)	0
	Moderate	4 (22%)	1 (20%)	7 (54%)
	High	1 (6%)	0	6 (46%)
KRT modality^#^	Not on KRT, wants KT	4 (22%)	0	-
	KT recipient, LD	2 (11%)^$^	0	-
	KT recipient, DD	3 (17%)	3 (60%)	-
	HD, waitlisted for KT	2 (11%)	0	-
	HD, does not want KT	4 (22%)	1 (20%)	-
	PD, waitlisted for KT	2 (11%)	1 (20%)	-
	PD, does not want KT	1 (6%)	0	-
Age at start of current KRT modality	All patients treated with KRT	68 (4)	-	-
	KT recipients	70 (6)	-	-
	Dialysis patients	67 (3)	-	-
Months on current KRT modality^#^	All KRT patients	14 (43)	8 (27)	-
	KT recipients	7 (23)	8 (44)	-
	Dialysis patients	19 (72)	8 (10)	-
Cause of ESKD	Cystic disease	3 (17%)	-	-
	Urological disease	2 (11%)	-	-
	TIN	2 (11%)	-	-
	Vascular	4 (22%)	-	-
	Other	4 (22%)	-	-
	Unknown	3 (17%)	-	-
Charlson Comorbidity Index	All patients	3 (1)	-	-
	KT recipients	3 (1)	-	-
	Dialysis patients	3 (1)	-	-
	Patients not on KRT	4 (1)	-	-
Profession	Nephrologist	-	-	5 (39%)
	Nurse	-	-	5 (39%)
	Medical social worker	-	-	3 (23%)
Clinical experience (years)		-	-	11 (4–42)

Quantitative data are presented as median (IQR) and categorical data as number of patients (percentage).

Abbreviations: DD = deceased donor; DM = diabetes mellitus; HD = haemodialysis; IQR = interquartile range; LD = living donor; PD = peritoneal dialysis; TIN = tubulo-interstitial nephritis.

^^*^^Educational level was categorised into low (no education, primary education or pre-vocational education), moderate (vocational education or selective secondary education) and high (university).

# For patients’ relatives: this displays the patients’ (time on) current KRT modality (none of the relatives had kidney disease themselves).

$ One patient pre-emptive.

Thematic analysis identified five themes. Table 2 shows these themes and the codes on which they are based. The five themes are described below and are illustrated by participant quotations (Table 3). Patients’ relatives agreed with patients in all cases and are therefore not described separately.

**Table 2:** Themes, categories and axial codes

Theme	Category	Axial code
1. KT and dialysis in older patients: attitudes and expectations	What is the best treatment?	KT is the best treatment, the one and only good choice; does not have any doubt about this.
		KT is not the best treatment modality for every (older) patient.
		KT is the best treatment, but not for every patient.
		The primary action is to establish if KT is possible.
	Attitude and expectations regarding KT	Patient chooses KT because they expect to be ‘normal’ again/have his/her normal life back after KT.
		Patient chooses KT because of the disadvantages of dialysis.
		Patient expects that KT will go well; is not afraid of it, is not worried about it.
		KT can have disadvantages.
		KT has many disadvantages and risks.
		The risks of KT are not worth the potential benefit.
	Attitude and expectations regarding dialysis	Patient considers dialysis a temporary solution (to survive) until KT is possible.
		Dialysis is a good treatment option.
		The negative image of dialysis is incorrect.
		Dialysis has advantages.
		Patient chooses dialysis because of disadvantages of KT.
		Dialysis can have many disadvantages, but not all patients experience these.
		Dialysis can have advantages.
2. KT or dialysis in older patients – Who makes the decision?	Who makes the decision?	The nephrologist decides if KT is possible.
		The patient decides whether or not they want KT (if the nephrologists considers KT possible).
		Relatives discuss the treatment options with patients, but leave the final decision to the patient.
		Nephrologist decides whether KT is possible.
		If KT is possible, the healthcare professional considers this the best treatment option, but the patient can make the final decision.
		In case of doubt regarding KT eligibility, healthcare professionals consult colleagues.
	Preferred level of patient-engagement	Patient wants to make (medical) decisions together with healthcare professionals.
		Patient wants to make all (medical) decisions him/herself.
		Patient leaves all (medical) decisions up to healthcare professionals.
		The choice between KT and dialysis becomes easier if the nephrologist states his/her preference.
		The (preferred) level of patient-engagement in (medical) decision-making differs per patient.
3. Assessment of KT eligibility: medical and personal values	Variables that participants use to asses eligibility for KT	*See Appendix 4*
	(Opinion about) key factors in KT eligibility assessment	Age should not be a contraindication for KT.
		Patient considers own medical history/comorbidity not relevant for eligibility assessment.
		Patient realises that medical history/comorbidity determines whether KT is possible or not.
		Eligibility for KT is determined by medical history and comorbidity.
		Patients are only eligible for KT if they are independent, therapy compliant, vital/in good physical condition and do not have significant cognitive impairment.
	Process of eligibility assessment	The eligibility assessment for KT should be objective, but is (at least partly) subjective.
4. Health outcome priorities	Health outcomes that participants consider while making the choice between KT and dialysis	*See Appendix 5*
	QOL	Patients consider QOL/feeling good the most important aspect in the decision-making process.
		QOL is an important health outcome.
		QOL cannot be measured objectively.
	Life expectancy	Patient does not consider life expectancy important in the decision-making process.
		Patient considers life expectancy important in the decision-making process.
	Preferences	Healthcare professional prefers to focus on objective measures.
5. Information provision and older patients’ needs	Informational needs of patients	Patient considers factual/medical information more important than stories of other patients.
		Patient considers stories of other patients more relevant than factual/medical information.
		Patient does not need to know all (medical) details.
		Patient wants to know all (medical) details).
	Information provision by HCP	Patients should receive all information, objectively
		Information provision is not purely objective.
		Information provision is adjusted to the patients’ educational level and cognitive status.
		Some patients are prepared (already know a lot about their treatment options) but other patients are not.

Abbreviations: HCP = healthcare professional.

**Table 3:** Participant quotations

Theme	Participant	Quote
1. KT and dialysis in older patients: attitudes and expectations	**Patients that prefer KT**	
	KT recipient (male, 69 years old, deceased donor)	‘I just wanted to have a normal life again. That was very important to me. It never occurred to me to consider either dialysis or a transplant. I just wanted to have a transplant, right from the start.’
	KT recipient (female, 66 years old, living donor)	(About KT) ‘I had no choice, I had to.’ Interviewer: ‘Well, you could have chosen dialysis instead, right?’ Patient: ‘No, I didn’t want that. (. . .) I would only have accepted dialysis to bridge the time until I would get a new kidney.’
	KT recipient (female, 66 years old, living donor)	(About experiences she heard from other KT recipients) ‘Afterwards, when they had received the transplant, they really were. . . It really felt like a miracle.’
	Patient not yet on KRT (female, 72 years old, looking for living donor)	(About why she wants KT) Patient: ‘I would be free again. I wouldn’t need to be on dialysis every other day, so I could do more! What I want is freedom. Not being dependent on others.’
	Patient not yet on KRT (male, 65 years old, waiting for deceased donor kidney)	(About KT) ‘I am not that afraid. Just do it, period. (. . .) It is like taking your car to the garage when it is broken. It has to be repaired and then the engine will run again.’
	HD patient (male, 70 years old, waiting for deceased donor kidney)	(About KT) ‘Yes, it can go wrong. But it can also go well. (. . .) I’ll just need to have a little faith in it. It won’t help me to think about what could possibly go wrong. That’s the way it is, and it can also turn out just fine.’
	KT recipient (male, 72 years old, deceased donor)	‘Dialysis is a necessary evil, it is a matter of survival’
	HD patient (female, 68 years old, waiting for deceased donor kidney)	Interviewer: ‘What are the advantages of KT for you?’ Patient: ‘Well, in any case, I will get rid of dialysis.’
	**Patients that prefer dialysis**	
	HD patient (male, 72 years old)	‘Right now, I’m doing well. Who knows what happens if I get a new kidney? Will I be as fit as I am now?’
	HD patient (male, 67 years old)	‘After KT, some risks will remain. (. . .) I am convinced it does bring a lot of benefits, but it also comes with some disadvantages in return. (. . .) I weighed the advantages versus the burden of the transplant trajectory. More specifically,: what will I get out of it and what will it cost me. For now, my conclusion is that a transplant would cost me too much.’
	HD patient (male, 73 years old)	(About KT) ‘I was already told there is a 95 percent chance of getting skin cancer. I don’t even remember everything that needs to be considered, my oh my. And what are the chances of rejection? (. . .) Apparently, you are lucky when the transplanted kidney works well. So, the way I feel now, I am definitely not undergoing a KT.’
	HD patient (male, 72 years old)	‘Starting dialysis was very good for me. (. . .) I feel more energised. Yesterday I dialyzed, and today I can just do anything I want.’
	PD patient (female, 81 years old)	‘(Dialysis) is just part of my daily life. It is normal for me.’
	**Healthcare professionals**	
	Nephrologist (male)	‘If a transplant is possible, it is a patient’s best option. However, if associated risks are estimated to be very high, it is the worst option.’
	Nurse (female)	‘I cannot make a general statement about whether dialysis or KT is the best choice, because it depends on the patient. But typically when I think of an active, vital person, I would lean towards offering transplantation. (. . .) KT in principle is superior, but not for everybody.’
	Nephrologist (female)	‘My immediate response is “is a transplant still possible”. Regardless of the patient’s age, this is actually the basic approach.’
	Nephrologist (male)	‘Donor kidneys are so scarce, both from deceased and living donors, that we have to be consciously use them. We cannot just give it a try. No, we have to have absolute faith that the patient is fully committed.’
	Medical social worker (female)	‘Some patients suffer from side-effects due to dialysis. They may experience “dialysis-hangovers”, not feeling well. They have less freedom.’
	Nurse (female)	‘In general, dialysis has a major impact on a patient. (. . .) It changes the patient’s life completely. (. . .) But I also do see older patients treated with dialysis three times per week without any problems. These patients did not feel too burdened and did well for a long period of time.’
	Nurse (female)	‘For some individuals, dialysis also provides social contacts. Older patients who are quite lonely might miss that after transplantation.’
2. KT or dialysis in older patients – who makes the decision?	**Patients**	
	HD patient (male, 76 years old, does not want KT)	(About the eligibility assessment for KT) ‘The doctors decide whether it is possible or not.’
	PD patient (female, 67 years old, waiting for deceased donor kidney)	‘I think I have to leave the risk assessment to the doctor. (. . .) Because as a patient, you don’t exactly know everything, for example, what the risks of the procedure are with certain cardiac conditions.’
	Patient not yet on KRT (male, 65 years old, waiting for deceased donor kidney)	‘If the doctor would decide that KT is not possible for me, I would have to accept that.’
	HD patient 4 (male, 73 years old, does not want KT)	(About the choice for dialysis instead of transplantation). Interviewer: ‘What did your doctor think of that?’ Patient: ‘Fine, of course. He accepted it. It’s my decision.’
	HD patient 3 (male, 67 years old, does not want KT)	‘The doctor is the specialist and can advise me, but I decide what I want. (. . .) A health care professional can only list the options.’
	PD patient (female, 81 years old, does not want KT)	‘I totally lack the knowledge, so yes, I will follow the doctors’ advice. (. . .) I always appreciate the doctor providing his opinion and view, because it gives me something to hold on to.’
	Patient not yet on KRT (male, 71 years old, waiting for deceased donor kidney)	Patient ‘I do not want to know about what can go wrong after KT.’ Interviewer: ‘But shouldn’t you know about the disadvantages too before you can make a decision?’. Patient: ‘Yes, that is why I leave that up to the doctor, that’s the specialist.’
	**Healthcare professionals**	
	Nephrologist (female)	‘If I decide that someone is not eligible for KT, I explain to the patient that he may have heard about transplantation. But a quite good physical condition is a prerequisite for that, which does not seem applicable for you. And that is usually enough.’
	Medical social worker (female)	‘If multiple options are available for a patient, we let the patient the treatment he prefers. However, sometimes certain treatment options are not medically possible and consequently the patient does not have a choice.’
	Nurse (male)	‘Nephrologists or other physicians may doubt if KT is possible. The nephrologist can then decide that KT cannot be done.’
	Nephrologist (female)	Interviewer: ‘To what extent can a patient decide to undergo KT or not?’ Nephrologist: ‘The patient can always say no. If someone doesn’t want it, than it is clear: the patient can say no.’
	Nephrologist (male)	‘When a patient decides not to have a transplant, this patient doesn’t choose the best treatment. (. . .) Obviously, for the physician, that doesn’t feel like the best treatment, and that causes some friction.’
	Nephrologist (male)	(About when a patient that is eligible for KT chooses dialysis instead) ‘I document that in medical files and letters. Because I’m afraid that colleagues who see this case, might ask “Why didn’t this woman receive a transplant? (. . .) What are you doing?”’
3. Assessment of KT eligibility: medical and personal values	**Patients and patients’ relatives**	
	HD patient (male, 73 years old, does not want KT)	‘If I was feeling bad, I would have opted for a kidney transplant. (. . .) However, I feel generally fine, so that is why I won’t do it.’
	HD patient (male, 72 years old, does not want KT)	‘I had a lot of medical problems last year and, well, I don’t want that anymore. So to be honest, that is why I don’t want to do it (KT). (. . .) And well, I am 72 years old and I can do what I want. That is why I say: those few hours on the dialysis ward, well, I just lie there quietly. Let them give that kidney to a younger person.’
	Patient not yet on KRT (female, 72 years old, waiting for deceased donor kidney)	(About the risks of KT) ‘I know I have a lot of medical issues, but I don’t see that as a risk. I have been living with those for 26 years.’
	PD patient (female, 66 years old, waiting for deceased donor kidney)	‘I can understand that if the heart is bad, it is not possible to undergo a KT.’
	HD patient (male, 76 years old, does not want KT)	(About why he considered the risks of KT too high) ‘I would get an old kidney, and my age, all those things. (. . .) And I’m thinking: give someone else this chance, a younger person, who deserves priority.’
	**Healthcare professionals**	
	Nephrologist (male)	‘If a patient is referred for KRT, I first examine (. . .) the medical history, looking for comorbidity, cardiovascular disease, (. . .) malignancy, (. . .) neurological disorders.’
	Nephrologist (female)	‘It depends on the severity of the comorbidities. A patient with severe heart failure, COPD and a high BMI, (. . .) may want KT, but that is out of the question. (. . .) Especially severe cardiovascular disease, such as heart failure or peripheral vascular disease, and also frailty, for example someone using a walker. With such a combination, I don’t expect someone to be better off with transplantation.’
	Medical social worker (female)	(About KT eligibility) ‘Heart and vasculature have to be decent, and cognition shouldn’t be too bad. That is the first “safety check” that comes to mind from my perspective as social worker.’
	Nephrologist (female)	‘In my opinion, life expectancy should be at least five years after KT and someone should be able to consciously enjoy life. (. . .) So, for someone with dementia in a nursing home, transplantation is not a logical option because of the life expectancy, the patient’s inability to consciously enjoy it and because of the costs for society.’
	Nephrologist (female)	‘In my opinion, a physician also has a certain responsibility to society. And yes, in case of scarcity, we have to try to allocate (organs) as fair as possible.’
	Nephrologist (female)	‘Whenever I see things go completely wrong for a patient after transplantation then, yes, I think I will tell the next patient to think about it carefully, because it is quite something. Yes, I am sure those experiences influence me.’
	Nurse (female)	‘Previous experiences play a role, I think. I have seen a lot and I know what the pitfalls are and what has the potential to succeed.’
4. Health outcome priorities	**Patients and patients’ relatives**	
	Patient not yet on KRT 1 (male, 65 years old)	‘I don’t want to throw myself into the misery of being stuck on peritoneal dialysis, because then I might as well be dead. (. . .) I would lose my freedom. Then I couldn’t do anything anymore. Such a life would be pointless for me. And I would impose it on my spouse as well.’
	Patient not yet on KRT (male, 71 years old, waiting for deceased donor)	(About life expectation after dialysis and KT) Patient: ‘We never spoke about that.’ (. . .) Interviewer: ‘Would you say you chose for living longer or for QOL?’ Patient: ‘Quality of course. (. . .) Being able to live, having fun.’
	KT recipient 4 (male, 74 years old, living donor)	Interviewer: ‘Why did you want the kidney transplant so badly?’ Patient: ‘Because I wanted to live to be 100 years old. (. . .) Having a new kidney, I would be able to live normally again. I would be able to eat and drink normally.’
	PD patient (female, 66 years old, looking for living donor)	Interviewer: ‘Why do you want KT?’. Patient: ‘Well, to have a normal life again.’
	Relative (male, 66 year old, partner of PD patient that wants KT)	‘She is not free to do what she wants. She has to lie on the bed for 9 h. She cannot get up when she wants because that requires disconnecting, which creates a lot of extra work. (. . .) The freedom that returns (after KT), that she can do what she wants. Not feeling tired all the time. (. . .) Just, living normally. Not connected to machines.’
	Relative (female, 31 years old, daughter of KT recipient)	‘We hoped he would receive the call that a kidney is available, so he would no longer need dialysis and get his live back. My parents have a caravan on a camping but they could not go there, because he was stuck to dialysis.’
	**Healthcare professionals**	
	Nephrologist (female)	Interviewer: ‘When do you consider a kidney transplant successful?’ Nephrologist: ‘When the patient gets through the first year relatively unscathed. The kidney function has to be good enough and last a few years, (. . .) and leave patients without severe infections. Unfortunately though, in older patients we quite often see really severe infections, and even death in the first year.’
	Nurse (female)	Interviewer: ‘What kind of older patient do you consider fit to receive a kidney transplant instead of dialysis?’ Nurse: ‘I would say, an older person that is physically active, moves vitally and without significant cognitive problems. An older person with a future, so to say.’ Interviewer: ‘What do you mean by “future”?’ Nurse: ‘Well, that you expect that they will live a few more years in good condition.’
	Medical social worker (female)	‘What I often see is that doctors prefer the treatment, dialysis or transplantation, which will extend the patient’s life.’
	Nephrologist (female)	‘As I said earlier, the first step is: is transplantation possible for this patient. Then I consider if the patient could physically handle it, or more specifically, will this patient live for the next five years in relatively good condition?’
	Nephrologist (male)	‘Would you rather live one year with excellent quality or fifteen years of moderate quality? You tell me. Maybe fifteen years with moderate QOL is superior. (. . .) It is a mix of QOL and life expectancy.’
	Nurse (male)	‘QOL is the most important outcome measure’
	Nephrologist (male)	‘I think it should be possible to select patient characteristics that can be used to assess the risk of complications, kidney function and QOL. I think the latter is the most important, especially for older patients. (. . .) However, we do not measure it, QOL. I think it might happen more and more in the future, but currently we do not really do that.’
	Medical social worker (female)	‘We always have to thoroughly interview patients about what “QOL” means to them. It is quite a general term. (. . .) For one patient, it mainly stood for the ability to maintain independence. Another patient described it as “I don’t want to be a patient”.’
5. Information provision and older patients’ needs	**Patients**	
	Patient not yet on KRT (male, 71 years old, waiting for deceased donor kidney)	‘I received piles of information booklets in the hospital. (. . .) I read those. (. . .) So, I was at least aware of the consequences. (. . .) Without it, a patient cannot have a meaningful conversation with a doctor.’
	KT recipient (male, 74 years old, deceased donor)	Interviewer: ‘What did you learn on the information evening?’ Patient: ‘They wrote down the advantages and disadvantages on a whiteboard. And that was about right, because afterwards I looked it up on the internet.’
	HD patient (male, 67 years old, does not want KT)	Interviewer: ‘Some people tell me that stories of other patients were very helpful to them. What do you think about that?’ Patient: ‘No. I did not feel the need. I that individual stories are too personal. Nobody is exactly like me and nobody is in the same situation as I am. So I don’t see the point of it.’
	Patient not yet on KRT (male, 65 years old, waiting for deceased donor kidney)	‘I don’t want to dwell on everything that might go wrong after a kidney transplant. I don’t read anything on the internet. (. . .) It has to happen, period.’
	Patient not yet on KRT (male, 66 years old, waiting for deceased donor kidney)	‘Of course, there are stories and information from doctors. But really learning about the experience itself. . . patients are the ones who are actually undergoing it. That will happen to me as well. That ís happening to me as well.’
	**Healthcare professionals**	
	Nephrologist (male)	‘Most important is that we properly inform and educate patients. (. . .) I try to do this objectively.’
	Medical social worker (female)	‘I want patients to have a realistic idea of all treatment options.’
	Nephrologist (female)	‘Of course we can influence patients a bit. I’m trying not to do this too much, but it happens naturally.’
	Nurse (female)	‘The treatment options are of course discussed with the nephrologist as well. I think patients take the nephrologist’s opinion into account in their decision, even unconsciously. Even if the doctors don’t want that, their opinion does count for the patient, especially for older patients.’
	Nurse (male)	‘Of course there are different types of patients. One will have higher education and another will only have completed primary school. I adjust my way of information provision.’

### Theme 1: KT and dialysis in older patients: attitudes and expectations

The attitude and expectations differed between patients preferring KT, patients preferring dialysis and healthcare professionals.

Patients preferring KT were convinced that KT is, without doubt, superior to dialysis. These patients choose KT because they expected to retain their ‘normal’ life after KT. After KT, they expected to be able to do what they want (e.g. hobbies, holidays, social contacts), to become more independent, to have more energy and/or to live longer. Some patients referred to KT as ‘a miracle’ or ‘a cure’ and could not mention any disadvantages of KT. Other patients acknowledged that KT has risks and that they would remain a patient for the rest of their life. However, all patients were convinced that they would not experience any (severe) complications or side effects. Their attitude towards dialysis was mainly negative. They expected (or experienced) that dialysis would have a negative impact on their daily life, QOL and/or life expectancy.

Patients preferring dialysis did not consider KT superior to dialysis. They felt that individual circumstances determine whether KT or dialysis is the best KRT modality for an older patient. Their expectations regarding KT were more negatively charged than patients preferring KT. For example, some dreaded the surgery or the use of immunosuppressive drugs. All of them mentioned multiple disadvantages of KT, such as the risk of infections or surgical complications, which they considered a significant risk for themselves. Regarding dialysis, these patients expected (or experienced) few dialysis-related restrictions or symptoms. In fact, they mentioned advantages of dialysis such as relieve symptoms since start of dialysis and the opportunity for social contacts they gained at the dialysis ward. Patients already on dialysis considered it as a normal part of their daily life.

Healthcare professionals considered KT the best KRT modality, also for older patients. However, healthcare professionals emphasised that KT has significant risks, and therefore not every patient is eligible for KT. Given these risks and the scarcity of donor kidneys, healthcare professionals did not consider KT as something to ‘just try’ for all of their patients. Similar to patients preferring KT, healthcare professionals predominantly described dialysis in a negative way. However, they were more nuanced and mentioned several advantages of dialysis, such as the possibility for frequent monitoring of the patient, which could increase medication adherence, and potential social contacts for patients. Moreover, all of them also knew dialysis patients who did not have many (medical) problems.

### Theme 2: KT or dialysis in older patients—Who makes the decision?

All participants reported that, in the end, the nephrologist decides whether a patient is eligible for KT or not. For this, patients trusted on the expertise of their nephrologist and most of them would accept it if he/she said that KT was not possible. For some nephrologists, this felt as a heavy responsibility because of the impact of their decision on their patients’ life. They frequently consulted other healthcare professionals, such as fellow nephrologists.

In addition, older patients preferred different levels of patient-engagement, which varied from ‘wanting to decide myself’ to ‘not wanting to decide at all’. Patients in the first group felt that they are the only one able to decide which treatment is best to them personally, whereas patients in the latter group felt that they lacked relevant (medical) knowledge, or they did not want to be responsible themselves. Healthcare professionals mentioned to be aware of these differences in preferred level of patient-engagement. Irrespective of patient preference, the majority of patients wanted to know the opinion of their nephrologist because they felt that this made the choice easier.

Nephrologists indicated that they feel somewhat uncomfortable, when an eligible patient decides to forego KT. Some nephrologists indicated that they try to convince such older patients to choose KT instead. However, all of them reported that the patient has the final say during this stage.

### Theme 3: Assessment of KT eligibility: medical and personal values

Appendix 4 shows an overview of all variables that healthcare professionals and patients reported to use for assessing eligibility for KT.

Healthcare professionals reported that eligibility for KT is mainly determined by medical history and comorbidity. In addition, assessment of the level of independence, therapy compliance, vitality/physical condition and cognitive status were essential factors during the eligibility assessment. Ethical factors, mainly related to organ allocation and a responsibility to the donor, were also considered and played a more prominent role than in younger patients. Nephrologists felt that a patient’s life expectancy should be long enough to justify the use of a scarce donor kidney. Nephrologists strived for objectivity in the eligibility assessment. However, all of them reported that, in reality, the eligibility assessment is not purely objective because of, for example, the influence of nephrologists’ personal values and previous experiences.

In contrast to healthcare professionals, medical factors played a less prominent role for the majority of the patients. For example, some patients did not consider their medical history (e.g. diabetes mellitus) to be relevant for the eligibility assessment. Instead, patients frequently used personal values to assess if KT would be possible for them. Examples of such personal values are their attitude towards KT and dialysis and whether they felt they deserved the kidney. Previous (medical) experiences also influenced the way patients assessed the risks of a treatment. For example, a patient that experienced severe complications after a previous surgery was very concerned about the KT surgery.

### Theme 4: Health outcome priorities

Appendix 5 displays all health outcomes that were considered by participants while making the choice between KT and dialysis. Although most health outcomes were mentioned by all participants, only healthcare professionals (not patients) mentioned ‘kidney function’ and ‘patient satisfaction’. Concerning the latter it is important to note that patients mentioned several other factors that will eventually result in (dis)satisfaction with the treatment. In addition, only patients (not healthcare professionals) mentioned ‘ability to perform caregiver duties’, ‘burden for relatives’, ‘feeling normal’, ‘feelings related to the presence of a donor organ’ and ‘freedom (being able to do what you want)’.

Healthcare professionals were quite unanimous in the way they valued health outcomes. They considered personal outcomes such as ‘QOL’ equally, or even more, important than medical outcomes such as ‘life expectancy’ and ‘surgical risks’. The input of medical social workers, who frequently discussed personal goals of care with patients, was highly valued. However, most healthcare professionals, including medical social workers, struggled with the fact that these personal goals of care frequently do not have clear definitions and cannot be measured objectively. As a consequence, in the actual decision-making phase, healthcare professionals mainly focused on medical outcomes.

The health outcome priorities of most older patients included QOL-related factors instead of general medical outcomes, such as life expectancy. Frequently mentioned health outcome priorities were ‘feeling normal’, ‘maintaining independence’ and ‘impact on daily life’. However, the goals of care differed greatly between individual patients. For instance, ‘life expectancy’ was considered the most important health outcome by some patients, whereas others did not consider ‘life expectancy’ relevant at all.

### Theme 5: Information provision and older patients’ needs

Healthcare professionals mentioned that they inform their patients in great detail about medical-technical aspects and risks of KT and dialysis. In addition to such factual, medical information, medical social workers also inform patients about more personal aspects, such as the impact of KRT on their (daily) life. They considered this information essential for patients to make a well-considered choice. Information provision was, if necessary, adjusted to the patients’ (expected) level of education and cognitive status. Most healthcare professionals wanted to provide objective information only. However, some of them acknowledged that their personal values, preferences and opinion influence the way they counsel patients.

This way of information provision seems suitable for some of the patients: our results show that some older patients indeed want to know all medical details. These patients also use other sources such as websites of patient federations to gain information. However, other patients have different informational needs. We found that some older patients were not interested in medical details at all. These patients felt that knowledge about all potential risks and disadvantages of a treatment would only make them (even more) worrisome. They considered the experiences of other patients far more valuable than (only) factual, medical information.

## Discussion

To our knowledge, this is the first qualitative study that explored the decision-making process regarding the choice between KT and dialysis in older adults. Our study shows that individual older patients report marked differences in prioritisation of health outcomes, the preferred level of engagement during the decision-making process and informational needs, while healthcare professionals were quite unanimous in their views. Older patients’ health outcome priorities were mostly related to QOL. Healthcare professionals also considered QOL-related outcomes important but found it difficult that they were neither objective nor measurable. Consequently, their focus tended to move towards more classical medical outcomes.

Previous research in older adults showed that, in general, older adults have different health outcome priorities than younger adults. For example, life expectancy is considered more important by younger adults (aged 21–60 years) than by older adults (aged >60 years) (19). Moreover, in community-dwelling frail older adults, goals of care are as often related to issues of well-being, such as living situation, social and family relationships, and mobility, as they are to medical problems (20). Similarly, many older adults with chronic kidney disease (CKD) prioritise QOL-related health outcomes, such as level of independence, above medical health outcomes, such as life expectancy (19, 21, 22). This highlights the importance of future research in the subgroup of older patients.

SDM in older adults differs from ‘regular’ SDM in two aspects: it is goal-oriented instead of disease-oriented (14, 15) and has different steps (14). After a short preparatory step, SDM in older adults should start with the identification of patient values and goals of care, and when discussing treatment possibilities, these factors should be taken into account. In other populations, such as patients with atrial fibrillation and older patients with cancer, explicitly discussing outcome priorities indeed provided new insights to healthcare professionals, and helped patients to make a treatment decision (23, 24).

Our study shows that, in the current decision-making process, patients, relatives and healthcare professionals collaborate and share their views. For instance, the majority of the patients and relatives reported that they wanted to know the treatment preference of their nephrologist. Moreover, healthcare professionals were motivated to inform their patients about all treatment options and also advised them during the actual decision-making phase. This collaboration is in line with the ‘collaborative deliberation’ model that describes interpersonal aspects that affect decisions and with the model of successful SDM in older adults (14, 25).

However, SDM in the decision-making process regarding the choice of KRT in older adults might be impeded by the discrepancy we observed between patients’ health outcome priorities (mainly QOL-related outcomes) and the variables that healthcare professionals reported to take into account during the decision-making process (mainly medical outcomes) (14, 26, 27). Although medical social workers explicitly discussed values and goals of care with their patients, these factors were not prioritised during the actual decision-making phase. The main reason for this finding, according to healthcare professionals, was that QOL-related outcomes are difficult to objectify.

Previous research already showed this tendency towards a disease-oriented approach by healthcare professionals (14). Moreover, clinical guidelines are frequently disease-specific and biomedically focused as well. Indeed, guidelines about decision-making recommendations in patients with ESKD mainly provide information about general medical outcomes, such as mortality and graft survival (2–4). Previous research also showed poor concordance between the health outcome priorities of patients with ESKD and healthcare professionals’ perceptions on these priorities (21, 28, 29). Thus, the decision-making process could benefit from early identification of older patients’ values and goals of care (in parallel with assessment of KT eligibility and before discussing the treatment options), and the explicit use of this information in further steps of the decision-making process.

This study has four limitations with potential impact on data saturation. First, we included only one patient with a high educational level. Educational level is associated with differences in patient involvement and needs during SDM (30). Socio-economic status, which is closely related to educational level, also influences doctor–patient communication (31). For example, patients from higher social classes receive a less directive and more participatory consulting style than patients from lower social classes (31). However, since the interviewed patients already presented a wide variety in personal values, prioritisation of health outcomes and preferred level of engagement during the decision-making process, we decided not to include additional patients with a high educational level. Second, we have only included participants from two hospitals and, in theory, inter-subject variation might be relatively small among participants from the same hospital. However, participants were recruited from one academic and one non-academic hospital, and they were successfully purposively sampled (e.g. on age, gender, KRT modality). A third limitation of this study is that patients already on dialysis and KT recipients might experience difficulties in recalling experiences regarding the decision-making process. However, we considered it valuable to include a wide variety of patients, including those that had completed the decision-making process. Lastly, data collection was not uniform: interviews were conducted face to face (*n* = 25), and, because of the COVID-19 pandemic, also via video call (*n* = 6) and by telephone (*n* = 5). However, previous research showed that face to face interviews are only marginally superior to video calling (32). Similarly, for interviews by telephone, there is no evidence of significant data loss, data distortion or reduced quality of the findings (33). This justifies the use of data collection via video calling and/or telephone, especially in case of limited time, budget, and/or access to people (32, 34).

This study meets all important quality criteria for qualitative research. First, we ensured that all possible perspectives were captured in our data by applying purposive sampling and by including all stakeholders involved in the decision-making process. We approached the issue from multiple perspectives (data triangulation) by including different types of patients (i.e. patients not yet on KRT, dialysis patients and KT recipients), while most other studies focused on the perspective of a single patient category (21, 35–38). Second, data collection and data analysis were parallel processes, which enabled exploration and verification of preliminary hypotheses during further data collection. Lastly, the influence of researchers’ personal characteristics and presumptions was minimised using two coders who independently analysed the data, and by frequently discussing the results during research meetings. The researchers involved in data analyses also have different personal characteristics, occupations and (clinical) experience, which contributes to investigator triangulation.

## Supplementary Material

aa-21-1938-File002_afac111
