# Supplementary material for: Kidney transplantation or dialysis in older adults—an interview study on the decision-making process
Source: Age Ageing. 2022 May 3;51(6):afac111. doi: 10.1093/ageing/afac111 (PMC9764842; doi:10.1093/ageing/afac111)
Supplement: aa-21-1938-File002_afac111 [file aa-21-1938-file002_afac111.docx]

**Appendices**

**Kidney transplantation or dialysis in older adults – an interview study on the decision-making process**

**Table of contents**

Appendix 1: Completed COREQ checklist Page 2

Appendix 2: Pre-dialysis and pre-transplant educational programs of participating centers Page 6

Appendix 3: Interview guide Page 8

Appendix 4: Variables that participants use to asses eligibility for KT Page 10

Appendix 5: Health outcomes that participants consider while Page 12

making the choice between KT and dialysis

**Appendix 1**. COREQ checklist

| **No** | **Item** | **Guide questions/description** | **Answers** |
| --- | --- | --- | --- |
| **Domain 1: Research team and reflexivity** | | | |
| Personal Characteristics | | | |
| 1. | Interviewer/facilitator | Which author/s conducted the interview or focus group? | TS and L. Huizinga (LHu) |
| 2. | Credentials | What were the researcher's credentials? *E.g. PhD, MD* | TS: MD  LHu: BSc |
| 3. | Occupation | What was their occupation at the time of the study? | TS: PhD candidate  LHu: medical student |
| 4. | Gender | Was the researcher male or female? | TS: female  LHu: male |
| 5. | Experience and training | What experience or training did the researcher have? | TS: Clinical experience (3 years resident internal medicine) and academic course qualitative research for PhD candidates.  LHu: Basic communication training during medical training and additional training for performing interviews in qualitative research. |
| Relationship with participants | | | |
| 6. | Relationship established | Was a relationship established prior to study commencement? | TS: No, expect for some of the participating nephrologists (via clinical work).  LHu: No. |
| 7. | Participant knowledge of the interviewer | What did the participants know about the researcher? e*.g. personal goals, reasons for doing the research* | All participants were unaware about personal goals, personal assumptions, and research interests of the interviewers. |
| 8. | Interviewer characteristics | What characteristics were reported about the interviewer/facilitator? e.g. *Bias, assumptions, reasons and interests in the research topic* |  |
| **Domain 2: study design** | | | |
| Theoretical framework | | | |
| 9. | Methodological orientation and Theory | What methodological orientation was stated to underpin the study? *e.g. grounded theory, discourse analysis, ethnography, phenomenology, content analysis* | Grounded theory approach |
| Participant selection | | | |
| 10. | Sampling | How were participants selected? *e.g. purposive, convenience, consecutive, snowball* | Patients and healthcare professionals: purposive sampling. Patients’ relatives: convenience sampling. |
| 11. | Method of approach | How were participants approached? e*.g. face-to-face, telephone, mail, email* | Face-to-face, by telephone or via email. |
| 12. | Sample size | How many participants were in the study? | 36 (18 patients, 5 relatives, and 13 healthcare professionals). |
| 13. | Non-participation | How many people refused to participate or dropped out? Reasons? | None of the approached subjects refused or withdrew participation. |
| Setting | | | |
| 14. | Setting of data collection | Where was the data collected? e*.g. home, clinic, workplace* | Home or clinic. |
| 15. | Presence of non-participants | Was anyone else present besides the participants and researchers? | No. |
| 16. | Description of sample | What are the important characteristics of the sample? *e.g. demographic data, date* | Interviews were performed from July to December 2020. Table 1 shows relevant demographic data. |
| Data collection | | | |
| 17. | Interview guide | Were questions, prompts, guides provided by the authors? Was it pilot tested? | Yes, see Appendix 2. The interview guide was not pilot tested. |
| 18. | Repeat interviews | Were repeat interviews carried out? If yes, how many? | No. |
| 19. | Audio/visual recording | Did the research use audio or visual recording to collect the data? | Yes, audio recording. |
| 20. | Field notes | Were field notes made during and/or after the interview or focus group? | Yes, after the interview, the interviewer wrote down what they thought was most remarkable about the interviewee’s answers and things that might be relevant during data analysis or further data collection. |
| 21. | Duration | What was the duration of the interviews or focus group? | Mean interview duration was 42 minutes (standard deviation 11 minutes). |
| 22. | Data saturation | Was data saturation discussed? | Yes, in frequent research meetings with TS, LHu, AK and MP. |
| 23. | Transcripts returned | Were transcripts returned to participants for comment and/or correction? | No. |
| **Domain 3: analysis and findings** | | | |
| Data analysis | | | |
| 24. | Number of data coders | How many data coders coded the data? | Two (TS and LHu). |
| 25. | Description of the coding tree | Did authors provide a description of the coding tree? | No. This is available upon request at the corresponding author. |
| 26. | Derivation of themes | Were themes identified in advance or derived from the data? | Derived from the data. |
| 27. | Software | What software, if applicable, was used to manage the data? | ATLAS.ti (version 8.4.20). |
| 28. | Participant checking | Did participants provide feedback on the findings? | No. |
| Reporting | | | |
| 29. | Quotations presented | Were participant quotations presented to illustrate the themes / findings? Was each quotation identified? e*.g. participant number* | Yes. |
| 30. | Data and findings consistent | Was there consistency between the data presented and the findings? | Yes. |
| 31. | Clarity of major themes | Were major themes clearly presented in the findings? | Yes. |
| 32. | Clarity of minor themes | Is there a description of diverse cases or discussion of minor themes? | There is a description of diverse cases. |

**Appendix 2**. Pre-dialysis and pre-transplant educational programs of participating centers

**Jeroen Bosch Hospital (JBZ)**

The nephrology ward of the JBZ has a special outpatient clinic pathway for patients nearing ESKD. Patients are referred to this pre-dialysis clinic when their eGFR falls below 15 ml/min/1.73m^2^ or, when there is a rapid decline, below 20 ml/min/1.73m^2^. A multidisciplinary team of healthcare workers (nephrologists, nurse practitioners, dialysis nurses, transplantation nurses, dietitians, and medical social workers) treats and educates patients about the different treatment options for ESKD: KT, hemodialysis (home and in-center), peritoneal dialysis, and conservative care.

At one of the first visits, the nephrologist mentions and briefly explains all treatment options to the patient. This is followed by two home visits of a medical social worker to discuss the impact of each treatment modality on the personal life of the patient. Thererafter the patient visits a nurse (practitioner), who extensively explains all treatment options. The oral explanation is supported by leaflets of the Dutch Kidney Foundation. Patients are also referred to the website of the Dutch Kidney Foundation, which contains personal stories of patients, information about dialysis and KT, and facts and figures (https://nierstichting.nl/leven-met-een-nierziekte/). Patient can also visit the dialysis ward and talk to dialysis patients. If necessary or desired by the patient, the (educational) session with the nurse or medical social worker is repeated. Patients also visit a dietician.

After the patient has visited all healthcare workers, a multidisciplinary meeting is organized in which a treatment advice is formulated. Subsequently, the nephrologist discusses this with the patient. In general, patients visit this outpatient clinic every 4-12 weeks. In February 2022, a total of 97 patients approaching ESKD were in follow-up at this outpatient clinic of the JBZ (55% aged ≥ 65 years old).

**Radboud University Medical Center (Radboudumc)**

The Radboudumc has a similar outpatient clinic pathway for patients nearing ESKD. Patients are referred to this clinic when their eGFR falls below 15 ml/min/1.73m^2^ or, when there is a rapid decline, below 20 ml/min/1.73m^2^. A multidisciplinary team of healthcare workers (nephrologists, nurse practitioners, dialysis nurses, transplantation nurses, dieticians, and medical social workers) treats and educates patients about the different treatment options for ESKD: KT, hemodialysis (home and in-center), peritoneal dialysis, and conservative care.

At one of the first visits, the nephrologist mentions and briefly explains all treatment options to the patient. This is followed by a visit to a nurse (practitioner), who will extensively explain all treatment options. The oral explanation is supported by leaflets of the Dutch Kidney Foundation. Patients are also referred to the website of the Dutch Kidney Foundation, which contains personal stories of patients, information about dialysis and KT, and facts and figures (https://nierstichting.nl/leven-met-een-nierziekte/). Patient can also visit the dialysis ward and talk to dialysis patients. If necessary or desired by the patient, the (educational) session with the nurse or medical social worker is repeated. For patients that are potentially eligible for KT, the Radboudumc organizes special information programs with presentations of a nephrologist, transplant nurse, medical social worker and kidney transplant patient. Patients can visit these programs together with their relatives. In addition to the visit to the nurse, patient also visit a medical social worker to discuss the impact of each treatment modality on the personal life of the patient. The medical social worker sometimes visits the patient at home. The patient also visits a dietician.

After the patient has visited all healthcare workers, a multidisciplinary meeting is organized in which a treatment advice is formulated. Subsequently, the nephrologist discusses this with the patient. These conversations and visits are repeated until the patient and nephrologist are able to make a final decision. In general, patients visit this outpatient clinic every 4-12 weeks. In February 2022, a total of 52 patients nearing ESKD were in follow-up at this outpatient clinic of the Radboudumc (52% aged ≥ 65 years old).

The Radboudumc also has an outpatient clinic for patients who are referred for KT (eligibility assessment) from other hospitals. In the consultations on this clinic, the focus lies on KT since patients will be (or have been) educated about other treatment options in their own hospital. A nephrologist and a transplant nurse extensively explain the risks and benefits of KT, in general and for the individual patient. In general, patients will visit this outpatient clinic once or twice, after which a decision about KT eligibility is made. All patients, including most eligible patients, will then be re-referred to their own hospital for follow-up until KT.

**Appendix 3.** Interview guide

(Translated from Dutch to English)

A) Patients

- How did you choose between dialysis and KT? Was this difficult? Did it feel like a choice? In the end, who made the decision?
- Why did you choose dialysis/KT? What did you hope/expect?
- Did you talk to other people about the choice between dialysis and KT?
- What did your doctor think was the best choice for you?
- Did you think you were eligible for KT?
- What do/did you think of KT/dialysis? What are advantages? What are disadvantages? What is the best treatment?
- What if the doctor said KT/dialysis was not an option for you?
- Do/did you expect your life will change after KT/dialysis? How?
- What did you know about dialysis/KT before you had kidney failure?
- How did you gain knowledge about dialysis/KT?
- Do/did you know someone treated with dialysis/KT?
- Do/did you receive information about dialysis/KT from healthcare professionals? What information was valuable to you?
- Are/were you prepared for dialysis/KT?
- How do you imagine your life will be in 5 years? Do you worry about the future? (e.g. life expectancy, living situation)
- To you, what is important in life? What are your life goals?
- How independent are you? How important is this for you?

B) Patients’ relatives

- How did your relative choose between dialysis and KT? Was this difficult? Did it feel like a choice? In the end, who made the decision?
- What was your role in the decision between dialysis and KT? Which treatment did you prefer?
- Why did your relative choose dialysis/KT? What did you hope/expect?
- What do/did you think of KT/dialysis? What are advantages? What are disadvantages? What is the best treatment?
- Are/were there things you hope your relative will be able to do or achieve in his/her life? Did this influence the decision between dialysis and KT?
- How did you support your relative in the decision-making process? And now?

C) Healthcare professionals

- How do you prepare for the conversation about the choice between KT and dialysis with an older adult?
- What factors are important to you when deciding between KT and dialysis in an older adult? (e.g. comorbidity, functional status, cognition)
- Is every patient eligible for both KT and dialysis?
- What are preconditions for KT?
- What role do ethical aspects, like organ allocation, play when deciding between KT and dialysis in an older adult?
- What are risks of KT and dialysis in an older adult? Have you treated patients with these complications? Does this affect your opinion about KT and dialysis in older adults?
- When do you consider KT and dialysis in an older adult successful?
- What part do life expectancy and quality of life play when deciding between KT and dialysis in an older adult?
- What is your role in the decision-making process? Who else are involved?
- In the end, who makes the decision between KT and dialysis in an older adult?
- What do you think of the role of the patient in the decision-making process?
- Are patients able to assess and value potential benefits and risks of KT and dialysis?
- What happens if the patients has another treatment preference than you?
- Do you always agree with colleagues regarding the choice between KT and dialysis in older adults?

**Appendix 4.** Variables that participants use to asses eligibility for KT

| **Baseline variable** (alphabetical order) | **Patients and their relatives** | **Healthcare professionals** |
| --- | --- | --- |
| Age | X | X |
| Attitude towards and expectations of KT and dialysis | X |  |
| Availability of donor kidney / expected waiting time | X | X |
| Biological age |  | X |
| Caregiver duties (e.g. care for spouse) | X |  |
| Cognitive status |  | X |
| Compliance |  | X |
| Costs |  | X |
| Current level of happiness | X |  |
| Expected quality of donor kidney | X |  |
| Experience with dialysis | X |  |
| Experience with previous medical problems (e.g. surgeries) | X |  |
| Feeling towards donor (e.g. afraid to fail guilty) | X |  |
| Feeling towards other patients on waiting list | X |  |
| Feeling towards receiving a donor organ | X |  |
| Frailty |  | X |
| Functional status (e.g. level of independence, activity level) |  | X |
| In dialysis patients: how dialysis is going |  | X |
| Life events (e.g. death of spouse) | X |  |
| Life goals | X | X |
| Lifestyle (e.g. smoking, drugs) |  | X |
| Medical history | X | X |
| Mental health (e.g. psychiatric disorders, coping mechanisms) |  | X |
| Perception of own health | X |  |
| Resilience |  | X |
| Risks for living donor |  | X |
| Scarcity of donor kidneys |  | X |
| Social situation |  | X |
| Social support | X |  |
| Symptom burden | X |  |
| Vitality |  | X |

**Appendix 5.** Health outcomes that participants consider while making the choice between KT and dialysis

| **Health outcomes** (alphabetical order) | **Patients and patients’ relatives** | **Healthcare professionals** |
| --- | --- | --- |
| Ability to perform caregiver duties | X |  |
| Burden for relatives | X |  |
| Dietary / fluid restrictions | X | X |
| Donor health (in case of living donor) | X | X |
| Duration of revalidation period after KT | X | X |
| Feeling normal | X |  |
| Feelings related to the presence of a donor organ | X |  |
| Freedom (being able to do what you want) | X |  |
| Graft survival/failure, rejection | X | X |
| Hospitalization rate | X | X |
| Impact on daily life | X | X |
| Kidney function |  | X |
| Level of independence | X | X |
| Life expectation (mortality/survival) | X | X |
| Malignancy | X | X |
| Number of drugs | X | X |
| Patient satisfaction |  | X |
| Quality of life | X | X |
| (Severe) infections | X | X |
| Side effects of (immunosuppressive) drugs | X | X |
| Surgical risks | X | X |
| Symptom burden | X | X |
